# The Launch of Annals of Occupational and Environmental Medicine

**DOI:** 10.1186/2052-4374-25-1

**Published:** 2013-05-21

**Authors:** Sang Baek Ko

**Affiliations:** 1 Editor-in-chief, Annals of Occupational and Environmental Medicine, Department of Preventive Medicine, Yonsei University Wonju College of Medicine, 162 Ilsan Dong, Wonju City, 220-701, Korea

## 

On behalf of the editorial board, I am pleased to announce the launch of a new international journal – *Annals of Occupational and Environmental Medicine* (AOEM). The AOEM is an open access, peer-reviewed, online journal that considers original research in occupational and environmental medicine. The online manuscript submission has been successfully implemented for the journal and is now open for new submissions at http://www.aoemj.com.

The AOEM is aimed at clinicians and researchers in the wide-ranging discipline of occupational and environmental medicine and health. The topics include but are not limited to the interactions between work and health, that is, subjects like occupational and environmental epidemiology, toxicology, hygiene, diagnosis & treatment of diseases, management, organization and policy.

The AOEM under its new name, will succeed *the Korean Journal of Occupational and Environmental Medicine* (KJOEM) as the official journal of the Korean Society of Occupational and Environmental Medicine (KSOEM). The KJOEM has been published only in Korean every year since 1989, started biannual publication in 1989, triannual publication in 1996, and quarterly publication in 1998. The KSOEM has endeavored to make the KJOEM a high-quality journal in occupational and environmental medicine mainly for researchers, workers, employers, and the government in Korea.

In line with recently changes in research boundaries and recommendations of the National Research Foundation (NRF) of Korea, we made the important decision to transform the KJOEM into an English language, high quality, international journal in occupational and environmental medicine. In order to meet the goal, we have created the Advisory Editorial Board of the AOEM, consisting of world-class, excellent and experienced professors and researchers in occupational and environmental medicine. We have also implemented a very efficient administrative system in the AOEM for streamlining the whole process from manuscript submission to on-line publication for authors.

I would like to thank my colleagues for their support and suggestions to make the launch of this new journal possible. I hope you will consider the *Annals of Occupational and Environmental Medicine* for your newest and most interesting research.

The author, Sang Baek Ko, has given permission for his photo to be used as the cover page.

